# How Monte Carlo heuristics aid to identify the physical processes of drug release kinetics

**DOI:** 10.1016/j.mex.2018.02.004

**Published:** 2018-03-02

**Authors:** Paola Lecca

**Affiliations:** Department of Mathematics, University of Trento, via Sommarive 14, 38123, Trento, Italy

**Keywords:** Heuristic algorithm based on Monte Carlo methods to simulate drug release profiles, Monte Carlo heuristics, drug release kinetics, drug release profile

## Abstract

We implement a Monte Carlo heuristic algorithm to model drug release from a solid dosage form. We show that with Monte Carlo simulations it is possible to identify and explain the causes of the unsatisfactory predictive power of current drug release models. It is well known that the power-law, the exponential models, as well as those derived from or inspired by them accurately reproduce only the first 60% of the release curve of a drug from a dosage form. In this study, by using Monte Carlo simulation approaches, we show that these models fit quite accurately almost the entire release profile when the release kinetics is not governed by the coexistence of different physico-chemical mechanisms. We show that the accuracy of the traditional models are comparable with those of Monte Carlo heuristics when these heuristics approximate and oversimply the phenomenology of drug release. This observation suggests to develop and use novel Monte Carlo simulation heuristics able to describe the complexity of the release kinetics, and consequently to generate data more similar to those observed in real experiments. Implementing Monte Carlo simulation heuristics of the drug release phenomenology may be much straightforward and efficient than hypothesizing and implementing from scratch complex mathematical models of the physical processes involved in drug release. Identifying and understanding through simulation heuristics what processes of this phenomenology reproduce the observed data and then formalize them in mathematics may allow avoiding time-consuming, trial-error based regression procedures.

Three bullet points, highlighting the customization of the procedure.

• An efficient heuristics based on Monte Carlo methods for simulating drug release from solid dosage form encodes is presented. It specifies the model of the physical process in a simple but accurate way in the formula of the Monte Carlo Micro Step (MCS) time interval.
• Given the experimentally observed curve of drug release, we point out how Monte Carlo heuristics can be integrated in an evolutionary algorithmic approach to infer the mode of MCS best fitting the observed data, and thus the observed release kinetics.
• The software implementing the method is written in R language, the free most used language in the bioinformaticians community.

## Graphical abstract

**Figure:**
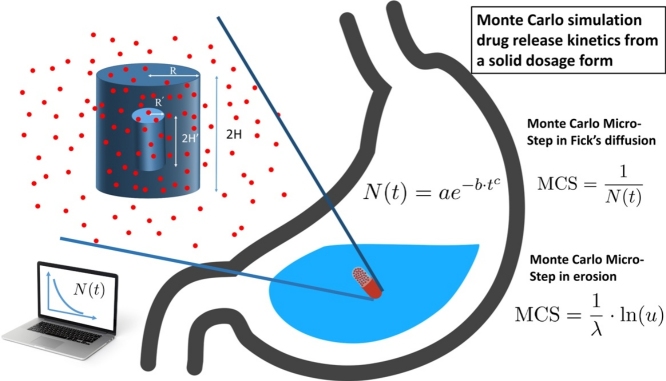

## Specifications Table

**Table:** 

Subject area	• *Biochemistry* • *Computer Science*
More specific subject area	*Chemoinformatics*
Method name	*Heuristic algorithm based on Monte Carlo methods to simulate drug release profiles*
Name and reference of original method	*The code presented in this paper implements Monte Carlo heuristics to simulate drug release from a cylindrical solid dosage form. The computational methods are inspired by the algorithms reported in the current literature and primarily in* P. Macheras, A. Iliadis, Modeling in biopharmaceutics, pharmacokinetics and pharmacodynamics: homogeneous and heterogeneous approaches, 2nd ed., Interdisciplinary applied mathematics Springer, Cham, Heidelberg, New York, Dordrecht, London, 2016
Resource availability	*The software implementing the Monte Carlo simulations has been written in R language* (www.r-project.com) *and the main R scripts are reported in Tables of this manuscript to allow the reproducibility of the results*

## Method details

**Fig. 1:**
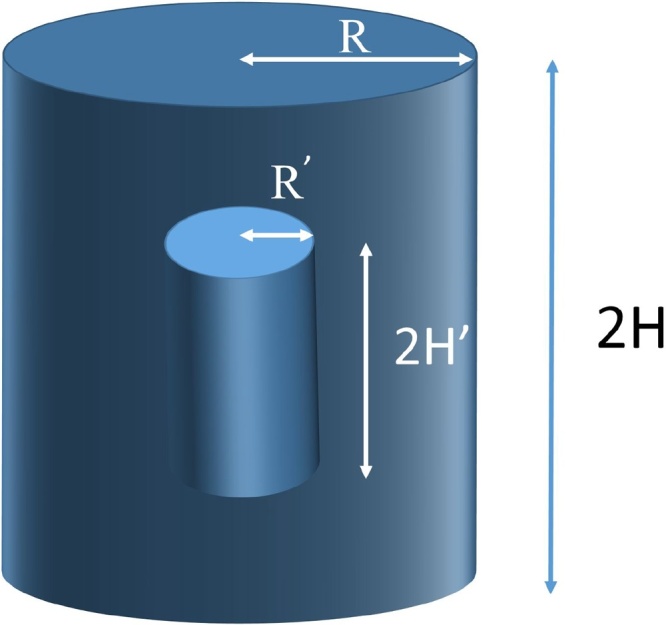
The drug transport with axial and radial release from a cylinder of height 2*H* and radius *R* is simulated as the shrinkage of the height and radius of the cylinder as the particles leave the release zone (adapted from Macheras and Iliadis [1]).

A drug in a solid cylindrical uniform dosage form is considered. Drug transport with axial and radial release from a cylinder of height 2*H* and radius *R* at time *t* is simulated. Drug release takes place from all sides of the big cylinder (Fig. 1). The drug mass is contained in the space between the big and the small cylinder. The molecules of the active substance of the drug are represented by random points uniformly distributed inside the cylinder. After time *t* the height of the cylinder becomes 2*H*’ and its radius becomes *R*’ (Fig. 1).

The simulation space is represented as a 3D lattice in the form of a cube. A homogeneous cylinder of radius *R* inside this cubic lattice is defined. The drug molecules occupy the space inside the cylinder, and each particle is represented by a cuboid *site* of unitary side. For simplicity, we assume that the cylinder is allowed to release drug molecules from its side surface, but not from its top or bottom, so that the height *H* remains constant over time. Moreover, we assume Fick’s diffusion of drug molecules and exclude volume interactions. The drug molecules are released in a dissolution medium (typically HCl [2]).

We simulate the release of drug molecules from the cylinder with a Monte Carlo algorithm consisting of the steps reported in Macheras and Iliadis [1] as follows.

1. A molecule is selected at random and moved to a randomly selected nearest-neighbour site.
2. If the new site is an empty site then the move is allowed and the molecule is moved to this new site. If the new site is already occupied, the move is rejected. A molecule is removed from the lattice as soon as it migrates to the leak site.
3. After each molecule move, time is incremented by arbitrary time units, called Monte Carlo Micro-Step (MCS) during which the movement takes place.

To begin, let us consider this definition

(1) $$MCS_{d}=\frac{1}{N\left(t\right)}$$

where N(t) is the number of drug molecules remaining inside the cylinder at time *t*.

According to this definition [3], since *N* decreases over time, the time step value increases over time. This means that the outflow of the active principle molecules is faster at the beginning than at the end of the simulation. The suffix “d” in Eq. (2) stays for “diffusion”, because as we will see later, this model of MCS step is indicative of a diffusive drug release mechanism.

A site is defined by its three coordinates (x,y,z). The sites are labelled as follows

a when for a site (*R* − 1)^2^ ≤ *x*^2^ + *y*^2^ ≤ *R*^2^, it is considered as a leak site
b if $x^{2}+y^{2}\leq {\left(R-1\right)}^{2}$, then the site belongs to the interior of the cylinder and it can host drug molecules
c if $x^{2}+y^{2}>{\left(R-1\right)}^{2}$, then the site is outside the cylinder, and it is marked as a restricted area, where particles are not allowed to go in.

As time passes, the radius *R* of the cylinder that ideally circumscribes the ​​space occupied by drug molecules narrows because of the leakage of molecules out of the leakage region. Consequently, at each simulation step, *R* is recalculated as the distance of the farthest point from the vertical symmetry axis of the cylinder, i.e.

(2) $$R\left(t\right)=\max_{i}\sqrt{x_{i}^{2}+y_{i}^{2}},i=1,\ldots ,N\left(t\right)$$

According to this representation of drug release, we indeed simulate a drug molecule that keeps moving until it gets to a site on the exposed border of the cylindrical matrix; once there, the molecule is immediately removed from the system and it is counted as drug released [3].

The definition of MCS is crucial to the simulation algorithm, because it determines how fast can drug molecules escape from the matrix-type release system, and consequently the analytical expression of the curve of N(t). It follows that it is precisely the definition of MCS that must contain all the variables explanatory of the actual physical processes of the drug release.

This procedure has been implemented in R language (www.r-project.org). The code consists of three main functions: **generate.points** (Table 1), **remove.points** (Table 2), and **escape.kinetics** (Table 3), implementing the generation of random points uniformly distributed in a cylindrical region and representing the drug molecules, the removal of points from the leakage region, and the computation of the number of drug molecules that remain inside the cylinder, respectively.

**Table 1:** This function generates a set of random points uniformly distributed in a cylindrical space. The function generates the point in such a way that their minimum inter-distance (the ”d” input parameter) can be chosen by the user. Moreover, the function calls the function “buffer.f” [4]. Buffer.f returns the original data table with buffered points removed.

**Table 2:** Function implementing the leak of drug molecules from the leakage region of the cylinder. The thickness of the leakage region is set to 1 (i.e. the size of the side of the cuboids partitioning the XYZ space)). The points that at time t belong to the leakage region are removed and then the radius of the cylinder is shrunk by one (ie. by the size of the cuboid side).

**Table 3:** Function that calls **remove.points** function to simulate the leakage of drug particles from the leakage regions and calculates the number of drug molecules remaining inside the cylinder.

The function **generate.points** generates a random angle *θ* ≤ 2*π*, a random *r* less than the radius of the cylinder, and a random *z* less than the height of the cylinder. In order to generate points with a given inter-distance between each other, the function **generate.points** calls a function **buffer.r** implemented by D.R. Roberts [4]. This function buffers points in XYZ space and returns the original data table with buffered points removed. It implements the following steps: 1) random selection of a single point, 2) removal of points within distance d of that point, and 3) random selection of the remaining points. **buffer.r** has to be ran numerous times, as the random point selection can result in more/fewer output points. The code of this function is shown in Supplementary information Section.

The function **remove.points** eliminates the points belonging to the leakage region (defined as the set of sites (x,y,z) such that ${\left(R-1\right)}^{2}\leq x^{2}+y^{2}\leq R^{2}$) and updates the size of the cylinder, and **escape.kinetics** simulates the release of drug particles (by calling **remove.points**), and calculates the time behaviour of the drug molecules remaining inside the cylinder. The cylindrical structure releases drug molecules at each simulation step and the algorithm updates the time variable *t* after each molecules’ leak by a quantity equal to the MCS.

**Fig. 2:**
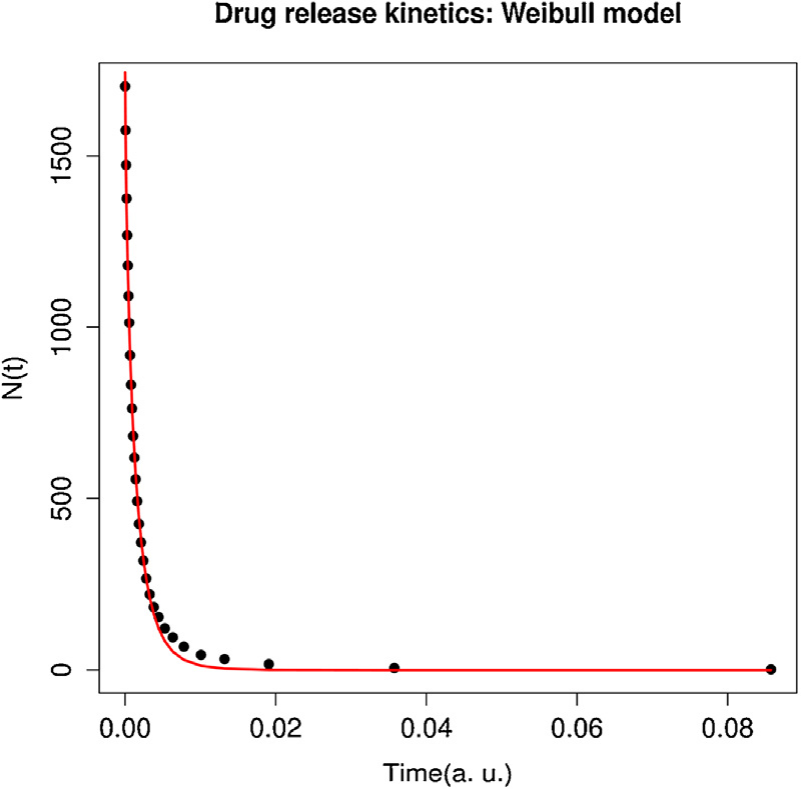
The red curve is the Weibull fit $\text{N}\left(\text{t}\right)=\text{a}\exp\left(-\text{b}\cdot {\text{t}}^{\text{c}}\right)$ to the data obtained with the Monte Carlo heuristics (black points). The estimates of the parameters of the Weibull function are reported in Table 4. The time is measures in arbitrary units (a. u.). (For interpretation of the references to colour in this figure legend, the reader is referred to the web version of this article.)

The release kinetics simulated by the Monte Carlo heuristics with MCS given in Eq. (1) (in arbitrary units) is fitted by Weibull model (Eq. (3)) [[5], [6], [3]], as shown in Fig. 2 and in Table 4.

(3) $$N\left(t\right)=a\exp\left(-b\cdot t^{c}\right)$$

**Table 4:** Parameters of the Weibull model obtained from a single simulation. Residual standard error: 22.75 on 28 degrees of freedom Algorithm “port” in R **nls** function (Non-Linear Least Squares fit).

Parameter	Estimate	Std. Error	t value	Pr(>|t|)	Signif.
*a*	1744.943	18.9815	91.929	<2e-16	0
*b*	160.2923	17.097	9.375	3.92E-10	0
*c*	0.7564	0.0171	44.23	<2e-16	0

**Fig. 3:**
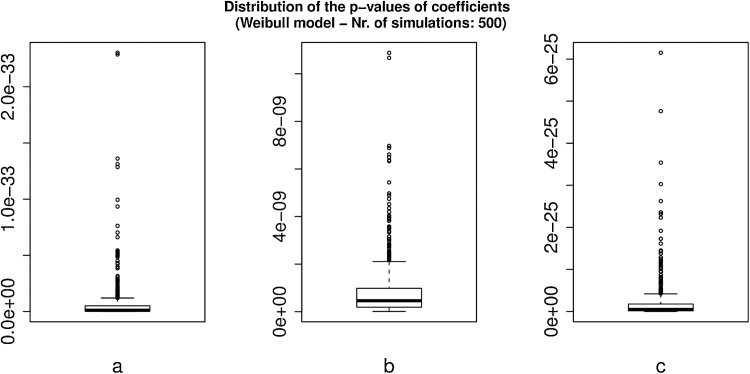
Boxplots of the distribution of the p-values for Weibull fit parameters a, b, and c in 500 simulations.

**Fig. 4:**
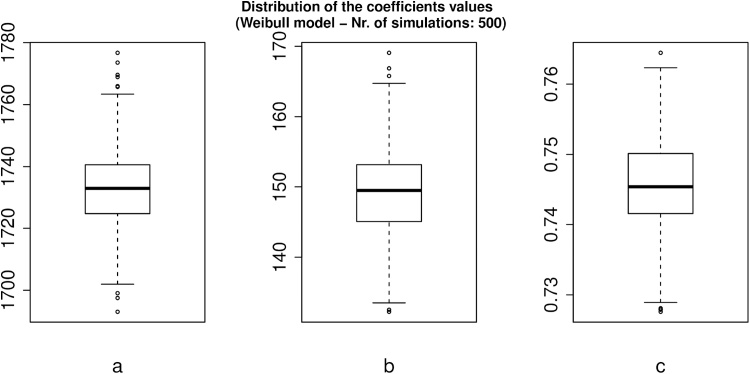
Boxplots of the distribution of the Weibull fit parameters a, b, and c in 500 simulations.

In Fig. 3, Fig. 4, we observe that the distributions of the values of the parameters of the Weibull model and of their p-values, obtained from 500 simulations, are very tight around the average value.

Since the simulations differ from each other by the different arrangement of points in space generated by the function **generate.points**, the parameters of the release curve vary from one simulation to another. Here, however, we see that the variation is small and that the Weibull model is a good fit for all simulations. In summary, these simulations show that the Weibull model describes nicely the entire drug release curve when the drug release mechanism is only Fickian diffusion [7].

If we consider a different mechanism of drug release, such as release by erosion of the polymeric matrix, a different model of the MCS time has to be implemented. Erosion represents the scission of polymeric chains, allowing solvent penetration into the leakage layer. On the microscopic scale, we can essentially look at this as a stochastic process, where the probability of chain breakage is a value following a known distribution. Such events occur independently of each other and at a specified rate within an interval of time

(4) $$MCS_{e}=\frac{1}{\lambda }\cdot \left|ln\left(u\right)\right|$$

where u∼U(0,1) is a random number from a uniform distribution and *λ* is the half-life time of the polymer (in units of time) that is characteristic of the material of the polymeric matrix [[8], [9]]. The suffix *“*e*”* in Eq. (4) stays for *“*erosion*”*.

The best fitting model for N(t), in case of release by erosion, is the linear one, i.e.

(5) N(t)=a+bt

**Fig. 5:**
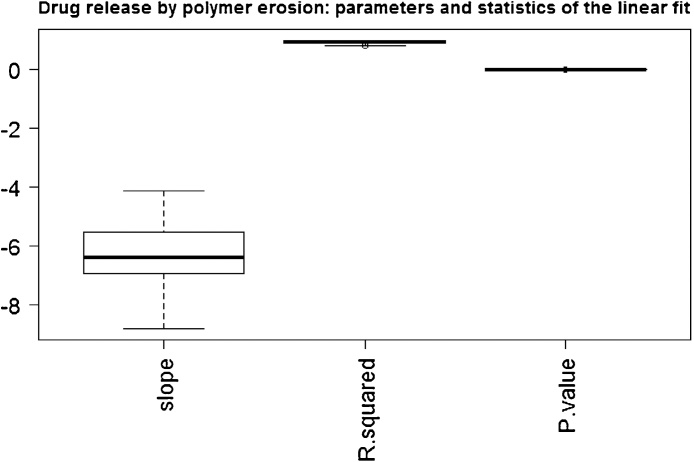
Slope, R-squared and p-value of the F statistic of the linear fit for N(t) given by Eq. (5) obtained in 50 simulation runs.

**Fig. 6:**
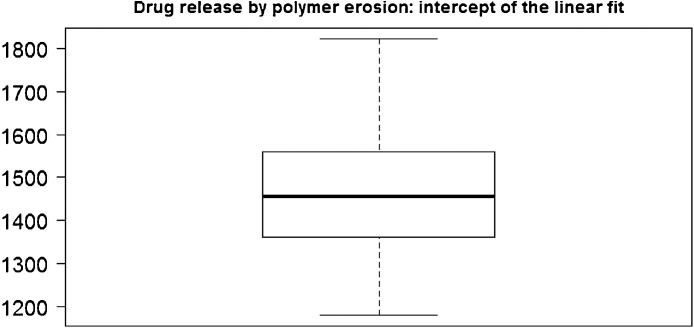
Distribution of the values of the intercept *a* of the linear fit for N(t) given in Eq. (5) obtained in 50 simulation runs.

**Fig. 7:**
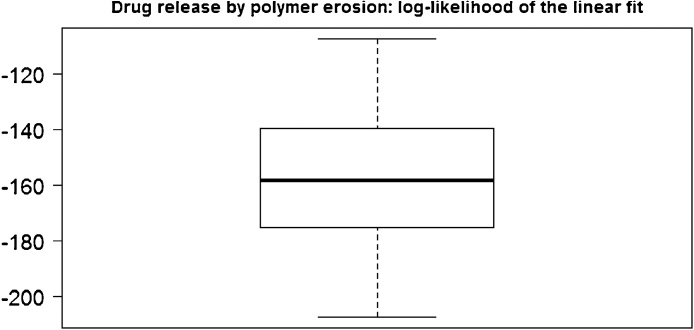
Distribution of the log-likelihood of the linear fit for N(t) given in Eq. (5), obtained in 50 simulation runs.

In Fig. 5, we show the distribution of the values of the slope (the parameter *b*), the *R*^2^ and the p-value of the F-statistics of the linear model obtained in 50 simulations run (with *λ* = 0.1). In Fig. 6, we show the distribution of the values of the intercept (the parameter *a*), and finally, in Fig. 7 we show the distribution of the log-likelihood of this model. These plots attest the goodness of the linear fit when the mechanism of drug release is the erosion.

The models of drug release by diffusion and by erosion we have discussed here are simplified representations of very complex scenarios described in the current literature [10]. Moreover, the erosion of the polymer containing the drug molecules is not the only mechanisms by which these molecules diffuse out the polymer.

However, the examples reported in this study highlight a noteworthy advantage of using Monte Carlo simulations as a general methodology applicable to more complex scenarios and instrument of inference of the complete release curve. The model of the MCS step discriminates the kinetics of the release. When MCS depends strongly on N(t), it is reasonable to suppose that the release kinetics is governed mainly by diffusion processes, whereas when MCS does not depend strongly on N(t), it is reasonable to suppose that stochastic effects due to complex random interactions at the micro-scale between the gastrointestinal fluids and polymeric matrix of the dosage form prevail on Fick’s deterministic diffusion. In general, the distinction between these two cases is not always clear, as stochastic effects may overlap with deterministic kinetics. A general, model for the Monte Carlo Micro-step is thus hybrid

(6) MCS=f(N(t),t)+ξ(Θ)

where *f* is a deterministic (noise-free) function of the time behaviour of the number of particles inside the drug. *ξ* is a noise term whose distribution is defined by the parameter vector Θ defining the characteristic of the polymeric matrix (e.g. the half-life time). Since the first term of MCS is a function of N(t), it is also an implicit function of the diffusion coefficient that governs the behaviour of N(t) and may be also space-dependent. Eq. (6) is thus a general “template” for the MCS definition, handling the parameters that are candidate to describe the mechanisms of drug release. In this study, we used

(7) $$MCS=A\cdot \frac{1}{{\left[N\left(t\right)\right]}^{\nu }}+B\cdot \xi \left(\Theta \right)$$

where the *A* and *B* are two time-dependent coefficients and *ν*∈ **R**. Therefore, the function *f* is defined as$f\left(N\left(t\right),t\right)=A\left(t\right)\cdot \frac{1}{{\left[N\left(t\right)\right]}^{\nu }}$.

Given an experimental release curve, Monte Carlo heuristics can be used in an evolutionary optimization approach to determine the parameters *A*, *B*, and *ν* of Eq. (7) that best reproduce the observation. The model of MCS inferred by such metaheuristic approach is informative of the physical processes governing the drug release.

In order to give some examples to support this statement, we show the models for the MCS in Eqs. (6) and (7) obtained with the Monte Carlo simulation heuristics described in this study and integrated in an evolutionary optimization procedure operating of real experimental data. Given the experimental release data points $\hat{N}\left(t\right)$, the estimation of the parameters of Eq. (7) is set up as an optimization problem to minimize the sum of squares ${\left(\hat{N}\left(t\right)-N\left(t\right)\right)}^{2}$. This optimization problem is solved using a genetic algorithm [11].

We used the data of drug release kinetics from hydroxypropyl methyl-cellullose (HPMC)-based pharmaceutical systems. An extensive literature reports significant experimental and theoretical work in revealing and modelling drug release from these systems. In particular, in this study, we used the experimental data reported by Siepmann et al. [2], Rinaki et al. [12], Bettini et al. [13], and Sung et al. [14].

These works studied the physico-chemical phenomena, which are involved in the swelling and drug release from hydrophilic matrix tablets. The hydrophilic carrier material used in the experiments was the hydrophilic methylcellulose (HPMC). The main characteristic of this material is its swellability. The biologic fluids water diffuse into the tablet, resulting in polymer chain relaxation with volume expansion. Then, the incorporate drug molecules disentangle from the sites of the devise and diffuse out of it [2]. Polymer swelling, drug diffusion, and drug and polymer dissolution are the key simultaneous processes governing the drug release kinetics from the HPMC tablet. The mathematical treatment of the drug release kinetics may become very complex, if not aided by heuristics simulations.

The hybrid stochastic/deterministic model for the MCS in Eq. (6), allows taking into account both the deterministic character of the diffusion processes and the inherently stochastic nature of the microscopic interactions between the biological fluids and the polymer molecular structure. The velocity with which the drug molecules disentangle from the polymeric matrix depends on the dissolution fluid and on the chain length and the viscosity of the HPMC used, but also on the rates of the reactions between fluid’s molecules and polymer molecules. These reactions are random events responsible for the stochasticity in the expression for the MCS in Eq. (6).

The experimental data we have used in this study, and the bibliographic reference of their authors are reported in Table 5. These data are representative of the behaviour of several other observed release kinetics, some of which also reported in Rinaki et al. [12] and Bettini et al. [13]. The drug release curve describes the fractional release amount of drug, i.e. the ration between the cumulative drug relased at time *t*, and the cumulative amount of drug released at time ∞.

**Table 5:** Experimental release data considered in this study concern HPMC cylindrical tablet of differnet viscosity, and two molecules, chlorpenoramine maleate and adynazolam mesylate, as drug-models.

Polymer	Drug	Reference
HPMC K15M	chlorphenoramine maleate	Siepmann et al. [2]
HPMC K4M	adynazolam mesylate	Sung et al. [14]
HPMC K15M	adynazolam mesylate	Sung et al. [14]
HPMC K100LV	adynazolam mesylate	Sung et al. [14]

**Fig. 8:**
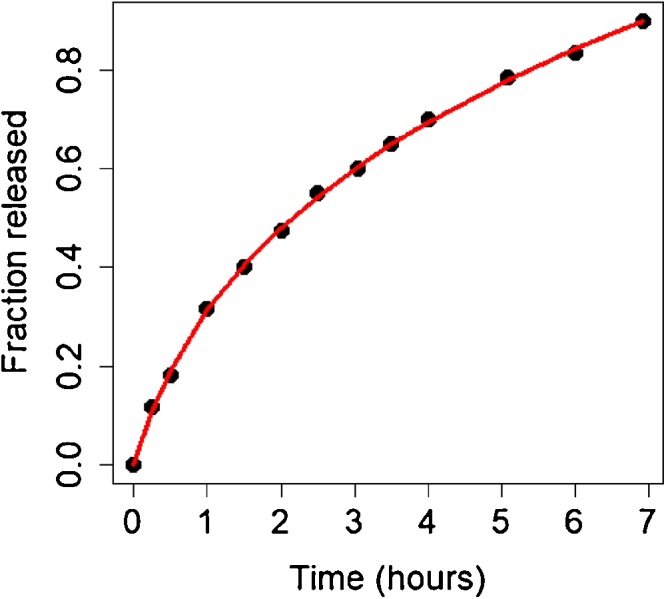
The experimental data points are accurately reproduced by a Monte Carlo heuristics, in which the predominant term in MCS is the deterministic one. Therefore, a Weibull model as defined in Eq. (3) (red curve) fits the entire release curve of chlorpheniramine maleate from HPMC K15M matrix tables (height 4 mm and height:radius ratio 1:1). The points are the experimental data reported in Siepmann et al. [2]. (For interpretation of the references to colour in this figure legend, the reader is referred to the web version of this article.)

We obtained that the experimental data of Siepmann et al. [2] are reproduced by Monte Carlo simulations where the radius *R* and the height *H* of the cylinder have length of 4 mm and the mass of the dosage form is 200 mg of chlorphenoramine maleate and 200 mg of polymer (for reducing the computational times, we set n = 200, so that one point in the 3D space contains 1 mg of substance). The MCS is calculated by Eq. (4). The fact that MCS that optimally reproduce the observations is expressed as the reciprocal of N(t) suggests that the predominant release mechanisms is the Fick diffusion. The model that fits the entire release curve is the Weibull model (Fig. 8 and Table 6).

**Table 6:** Parameter of the Weibull model (Eq. (3)) obtained in an individual simulation run fitting the release data in Fig. 8 [2]. Residual standard error: 0.006114 on 10 degrees of freedom. The fitting algorithm is “port” in the **nls** function of R.

Parameter	Estimate	Std. Error	t value	Pr(>|t|)	Signif.
*a*	8.0121	2.1033	3.809	0.00343	0.001
*b*	3.2424	0.2618	12.386	2.17e-07	0
*c*	−0.2033	0.0197	−10.318	1.19e-06	0

**Fig. 9:**
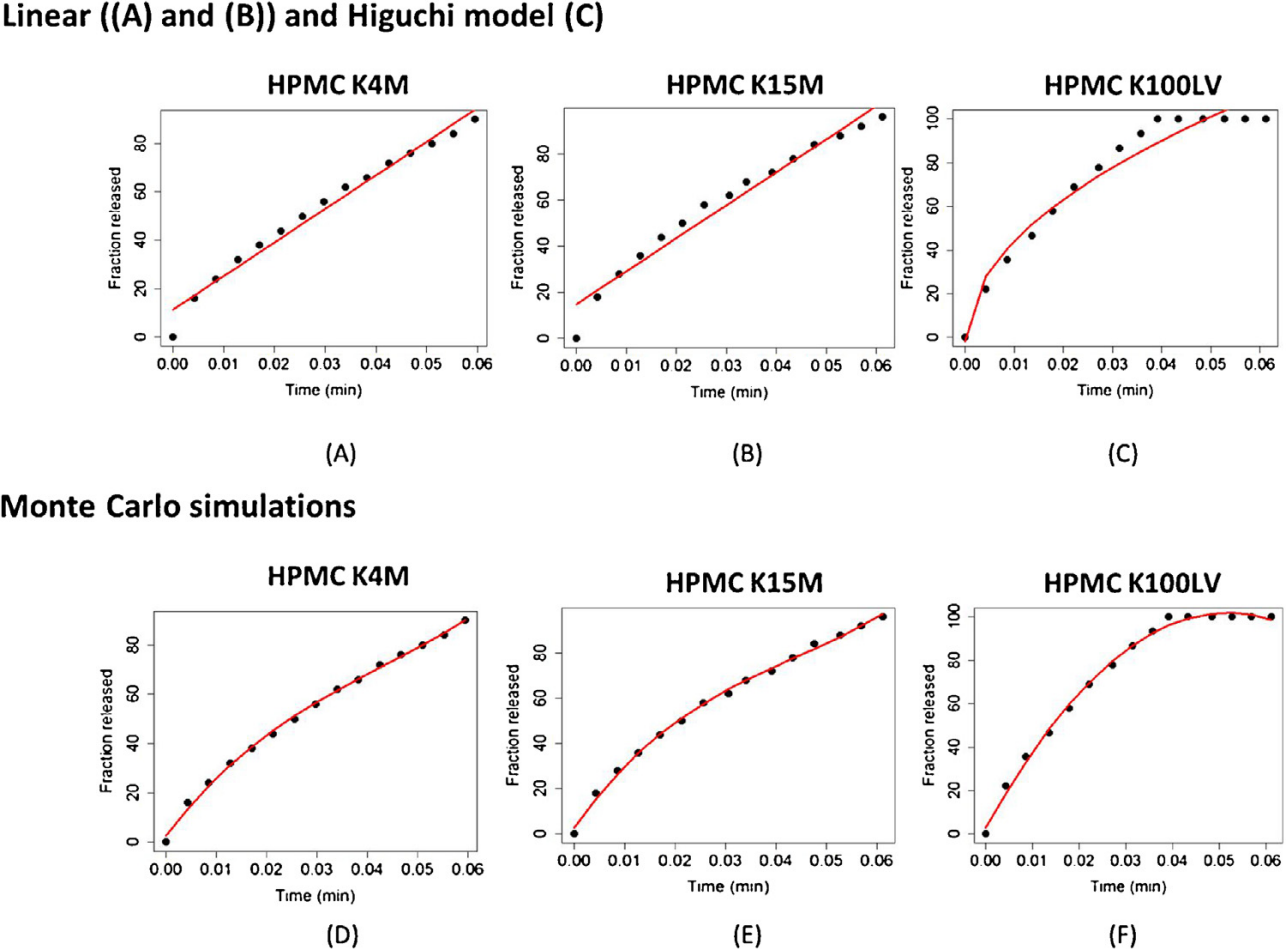
Fitting of adinazolam mesylate release data from HPMC matrices of different viscosity [14]: HPMC 4M, HPMC K15M, and HPMC 100LV). The (A), (B) and (C) plots show the regression of the experimental data with linear models ((A) and (B)) and Higuchi model (C). The Weibull model does not give a convergent fit in any of three cases. Linear models and the Higuchi model fit the data points only partially. Average Monte Carlo simulation curves from 50 simulation runs ((D), €, (F)) fits more accurately the data points and reveal the coexistence of different release mechanisms (see the text).

The model fitting the data of Sung et al. are more complex. In Fig. 9, we see that best, but nevertheless sub-optimal fit to the experimental points are the linear and the Higuchi model (∝ √ *t*). Indeed, these models fit only partially the experimental data points. Using Monte Carlo approach, we obtained that the MCS expression at the simulation step *k* that is able to reproduce more accurately the release curve has the following form:

(8) $$MCS=\Delta t_{k+1,k}=t_{k+1}-t_{k}=A\left(k\right)\frac{\lambda_{1}}{N\left(t_{k+1}\right)}+B\left(k\right)\frac{1}{\lambda_{2}}|\ln\left(u\right)|$$

where $A\left(k\right)=\frac{1}{k},B\left(k\right)=k$. The coefficients *λ*_1_ and *λ*_2_ assume different values for polymer of different viscosities. The simulation have been performed for a cylindrical tablet with radius and height of length 4 mm, and 200 mg of drug and 200 mg of polymer.

The plots (D), (E), and (F) of Fig. 9 show that the Monte Carlo heuristics with the MCS given by Eq. (8) fit the experimental data much more accurately than the usually adopted linear and Higuchi models.

The model of MCS in Eq. (7) reveals that the release kinetics observed by Sung et al. [14] might be governed by different processes over different time intervals. When k is small (at the beginning of the release), the deterministic diffusion term contributes significantly, whereas when k is larger the deterministic contribution becomes smaller. Observing the Monte Carlo simulation curves in (D), (E) and (F) plots of Fig. 9, we can see that the first 60%–70% of the release curve is convex and the remaining 40%–30% is a straight line or approximately a plateau. Indeed, the experimental data themselves exhibit different kinetics faithfully mimicked by two different behaviors for the simulation curve. Monte Carlo simulations suggest that the first 60%–70% of the release curve the diffusion may be the predominant mechanism, whereas in the last 40%–30% more complex stochastic phenomena due to the polymer swelling are predominant.

In summary, the coexistence of different physical mechanisms that determines the release kinetics often makes it difficult to find a curve model of the observed data through conventional regression procedures. A Monte Carlo algorithm like the one presented in this study based on a hybrid model for MCS optimized on experimental data can help in identifying the nature of the release mechanisms and the prevalence of one or the other throughout the entire observation period. This facilitates the construction of a mathematical model for the observed release data points.
