# Supplementary material for: How Monte Carlo heuristics aid to identify the physical processes of drug release kinetics
Source: MethodsX. 2018 Mar 2;5:204–16. doi: 10.1016/j.mex.2018.02.004 (PMC5959741; doi:10.1016/j.mex.2018.02.004)
Supplement: Supplementary file 1 [file mmc1.docx]

**Supplementary material *and* additional information:**

Table 1S reports the function **buffer.f** used in this study**.** This is an extension to the three-dimensional case of the **buffer.f** function originally implemented by David R. Roberts [4] and available online at <https://davidrroberts.wordpress.com/2015/09/25/spatial-buffering-of-points-in-r-while-retaining-maximum-sample-size/>

**Buffer.f** implements the following steps:

1) random selection a single point,

2) removal of points within distance d of that point,

3) random selection of the remaining points.

To obtain results that are more robust, numerous iterations of this function has to be ran, as the random point selection can result in a different number of output points.

In our code we used the function in Table 1S, because from a set of points in XYZ space, we want to retain only those points at least a given distance **buffer=d** from one another. Furthermore, in removing points that do not satisfy this requirement, we want to keep as much points as possible.

Table 1S: the function returns the original data matrix of the points in 3D space with buffered points removed. This function is a modified version of the function available in [4] (see the text).

**## Function to buffer points in XYZ space:**

**# Inputs:**

**# original.data.points - a data.frame to select from with columns x, y, z**

**# buffer - the minimum distance between output points**

**# reps - the number of reps for the points selection**

**buffer.f <- function(original.data.points, buffer, reps){**

**# List of vectors**

**suitable <- list()**

**for(k in 1:reps){**

**# output vector**

**outvec <- as.numeric(c())**

**# vector of buffered out points**

**bufferedvec <- c()**

**for(i in 1:nrow(original.data.points)){**

**# Stop running when all points exhausted**

**if(length(bufferedvec)<nrow(original.data.points ))**

**{**

**# Set the rows to sample from**

**if(i>1){**

**rowsleft <- (1:nrow(original.data.points))[-c(bufferedvec)]**

**} else {**

**rowsleft <- 1:nrow(original.data.points)**

**}**

**# Randomly select point**

**outpoint <- as.numeric(sample(as.character(rowsleft),1))**

**outvec[i] <- outpoint**

**# Remove points within buffer**

**outcoord <- original.data.points[outpoint,c("x","y","z")]**

**bufferedvec <- c(bufferedvec,**

**which(sqrt((original.data.points$x-outcoord$x)^2**

**+ (original.data.points$y-outcoord$y)^2**

**+ (original.data.points$z-outcoord$z)^2)**

**<buffer))**

**# Remove unnecessary duplicates in the buffered points**

**bufferedvec <- bufferedvec[!duplicated(bufferedvec)]**

**}**

**}**

**# Populate the suitable points list**

**suitable[[k]] <- outvec**

**}**

**# Go through the iterations and pick a list with the most data**

**best <- unlist(suitable[which.max(lapply(suitable,length))])**

**original.data.points[best,]**

}
